# Using Artificial Neural Networks to Predict Influences of Heterogeneity on Rock Strength at Different Strain Rates

**DOI:** 10.3390/ma14113042

**Published:** 2021-06-03

**Authors:** Sheng Jiang, Mansour Sharafisafa, Luming Shen

**Affiliations:** School of Civil Engineering, The University of Sydney, Sydney, NSW 2006, Australia; sheng.jiang@sydney.edu.au (S.J.); mansour.sharafisafa@sydney.edu.au (M.S.)

**Keywords:** rock-like material, heterogeneity, artificial neural networks, 3D printing, strain rate, strength

## Abstract

Pre-existing cracks and associated filling materials cause the significant heterogeneity of natural rocks and rock masses. The induced heterogeneity changes the rock properties. This paper targets the gap in the existing literature regarding the adopting of artificial neural network approaches to efficiently and accurately predict the influences of heterogeneity on the strength of 3D-printed rocks at different strain rates. Herein, rock heterogeneity is reflected by different pre-existing crack and filling material configurations, quantitatively defined by the crack number, initial crack orientation with loading axis, crack tip distance, and crack offset distance. The artificial neural network model can be trained, validated, and tested by finite 42 quasi-static and 42 dynamic Brazilian disc experimental tests to establish the relationship between the rock strength and heterogeneous parameters at different strain rates. The artificial neural network architecture, including the hidden layer number and transfer functions, is optimized by the corresponding parametric study. Once trained, the proposed artificial neural network model generates an excellent prediction accuracy for influences of high dimensional heterogeneous parameters and strain rate on rock strength. The sensitivity analysis indicates that strain rate is the most important physical quantity affecting the strength of heterogeneous rock.

## 1. Introduction

Natural rocks typically have substantial heterogeneity in terms of containing pre-existing flaws at multiple scales resulting from a variety of geological processes. The heterogeneity makes alternations to rock mechanical strength, further affecting the deformation behaviors and failure patterns upon loading [1,2]. In fact, to a certain degree, almost all rock-related engineering projects include structure constructions in rock masses, which involve pre-existing opening cracks [3,4]. For example, human excavations can greatly alter the stability of rock masses as a result of disturbance of the primary stress equilibrium, leading to artificially induced stress redistribution. Consequently, stress concentration may occur around the pre-existing flaws, which triggers crack coalescence and ultimate failure [5,6]. These opening flaws are usually naturally filled with different fine-grained materials due to weathering or joint shearing [7]. In geological constructions, opening flaws are manually filled via grouting and shotcrete to strengthen the load capacity of flawed rocks [8,9]. The addition of filling materials further enhances the rock heterogeneity. Fracture behaviors of flawed rock become more complex with the involvement of filling materials due to the complicated interactions between rock and flaw filling. In addition, natural rocks are often subjected to high strain rate loadings, such as earthquakes, rock blasting and oil-well fracturing. As a rate-dependent material, the heterogeneous rock mechanical strength is also highly related to the strain rate [10].

Hence, it is vital to establish the relationship between the heterogeneity on the flawed rock (induced by pre-existing flaws and filling materials) concerning the mechanical strength at different strain rates, bringing great convenience to rock engineering application and geotechnical design. In terms of evaluating the rock mechanical strength, laboratory tests are the most widely applied direct approaches, where standardized specimens are indeed required according to the corresponding testing standards. So far, several laboratory experiments have been performed on the flawed rocks with different pre-existing crack configurations, such as crack geometry, number, orientation, etc. [11,12]. For instance, by performing static and dynamic experiments on cubic rock specimens with a single pre-existing flaw, Yan et al. [13] illustrated the significant coupled effects of pre-compression and strain rate on rock strength and fracture responses. Zhao et al. [14] investigated the cracking and coalescence modes on the rock-like specimen containing two parallel cracks via uniaxial compression tests. They highlighted that the peak strength of the tested samples is related to the crack inclination angle and the associated bridge angle. In addition, Huang et al. [15] put more emphasis on probing into the effects of confining pressures. Regarding three pre-existing fissures, Zhou et al. [16] focused on the investigation of coupled thermomechanical and underlying cracking mechanisms. However, the major drawbacks of experiments are also obvious; they are known to be time-consuming and expensive. Considering the multiple heterogeneous parameters and strain rate that influence the mechanical behaviors of the flawed rock, a large number of samples with variations are required. For some special fragile or heavily weathered rocks, an adequate number of high-quality formed samples is not always feasible to obtain. Therefore, laboratory-based rock mechanical strength estimation may be problematic, especially for investigating high dimensional variables.

An alternative approach is to run numerical simulations by performing parametric studies to reduce the excessive experimental workload and cut the unnecessary cost [17,18]. For example, Wang et al. [19] performed extended finite element method simulation to investigate the initiation mechanisms of wing cracks, which stayed consistent with the theoretical analysis. Liu et al. [20] incorporated a fluid coupled model into grain-based model to discover the promotion mechanisms for crack coalescence in two systems with pre-existing flaws. Shen et al. [21] adopted a discrete element method to establish the relationships between P wave velocity and the number of pre-existing cracks, as well as confining pressure. However, as an indirect approach, the simulation study has its inherent disadvantages. Before conducting simulation, a thorough understanding is needed, with an awareness of all the factors involved. Meanwhile, a considerable amount of time is spent on validating the material constitutive parameters. Other conventional method is either seeking an analytical solution or obtaining the empirical solution for simplicity and reliability [22]. Nevertheless, these two options are not always achievable for complicated problems involving nonlinear and high-dimensional variables [23].

Recently, the artificial neural network (ANN) approach has shown promising capabilities in handling the abovementioned complex problem [24,25]. The ANN-based approach presented more reliable and acceptable results than conventional statistical analysis, as reported in [26]. ANN has an inherent ability to learn from available datasets and has been proven to be a powerful tool in various fields. For example, Gope et al. [27] designed an efficient ANN architecture to predict the crack growth direction of aluminum alloys. Liu and Athanasiou [23] successfully captured the complex relationship among the plane–strain stress intensity factor, the indentation load, and the specimen dimensions through ANN. Yan et al. [28] proved that ANN models are viable for predicting fracture parameters with high accuracy by using only 77 sets of experimental data. For predicting the rock strength, the previous ANN models primarily take the inherent physical properties of rock materials into account, e.g., porosity [29], P wave velocity [29], unit weight [30] and point load index [29,30]. Some of the input parameters considered, such as lithology, are only qualitatively introduced into the neural network [30]. However, limited attention was paid to forecasting the influences of the rock heterogeneity on rock strength by the ANN approach. In addition, the strain rate effect was also ignored in the previous ANN-based models despite its importance.

Targeting the gaps in the existing literature, this study adopts the ANN approach to predict the influences of heterogeneity on rock strength at different strain rates. The ANN model is implemented in Matlab (R2020a). The raw dataset is collected from our previous 84 Brazilian disc tests [31,32,33]. In these tests, 3D-printed Brazilian disc specimens with pre-existing single and double flaws were compressed under quasi-static and dynamic loadings. The application of the 3D printing technique can eliminate the creation of micro cracks and guarantee the imprecise flaws in the geometry during the flaw embedding process, making the experimental results more reliable. The considered heterogeneous parameters in this study include the flaw number, relative flaw location, flaw orientation, and strain rate. The optimization of the ANN architecture is performed using a trial-and-error method in terms of transfer functions and hidden neuron numbers. The predictions obtained from the trained ANN model are compared with the experiments to evaluate ANN model performance. In addition, a sensitivity study demonstrates the relative importance of the individual input variable, and the effects of different input numbers are also investigated.

## 2. Methodology

**Figure 1:**
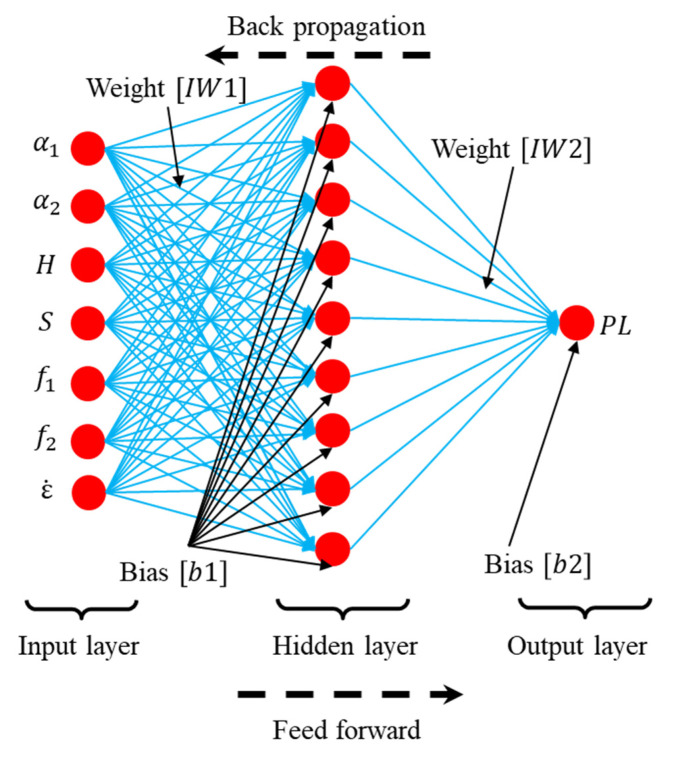
The architecture of an artificial neural network containing a single hidden layer.

As a versatile approach for dealing with nonlinear problems, the artificial neural network (ANN) is in the form of a multilayer network, consisting of two propagation directions, namely feed forward and back propagation [24]. As shown in Figure 1, a typical ANN architecture is divided into three parts: one input layer, one or multiple hidden layers, and one output layer. Multiple layers containing different numbers of neurons are connected by either nonlinear or linear transfer functions. The complex nonlinear relationship between input and output variables can be learned via these transfer functions. The collection of neuron nodes distributed in each layer has their unique weight and bias values to describe the interconnection strength within the network. In Figure 1, [IW1] and [IW2] represent the input weight matrixes, while [b1] and [b2] represent the bias matrixes in the hidden and output layers, respectively.

Herein, the updating of the weight and bias matrixes is achieved by adopting the Levenberg–Marquardt algorithm as the training function. As a typical backpropagation algorithm, the Levenberg–Marquardt optimization has its inherent advantages, pertaining to convergence rate, generalization performance and precision, although more memory is required [34]. Since the Levenberg–Marquardt algorithm is an iterative procedure, the application of training function is a process of adjusting the network weight and bias matrixes for each epoch. The initial weight and bias values are random, and there is no need to make a prior assumption. The algorithm implementation has two phases. Firstly, input variables are propagated forward through the network via transfer functions to emerge as output variables, defined as a feed-forward process. To assess the ANN model performance, the averaged squared difference between the outputs and targets is proposed and quantified by a mean square error (*MSE*):

(1) $$MSE=\frac{1}{n}\overset{n}{\underset{i=1}{\sum }}{\left(O_{i}-T_{i}\right)}^{2}$$

Meanwhile, the correlation between the outputs and targets is expressed by a regression value R as:

(2) $$R^{2}=1-\frac{\sum_{1}^{n}{\left(O_{i}-T_{i}\right)}^{2}}{\sum_{1}^{n}O_{i}^{2}}$$

where *n* is the number of outputs, and $O_{i}$ and $T_{i}$ are the *i*th output and target values, respectively.

During training, if the *MSE* meets the target error, the training process is then terminated because the predictive accuracy is sufficient. Otherwise, the error between the predicted output and target output values will be propagated backward through the network to update the connection weights and biases, in order to minimize the errors, until the *MSE* satisfies the target error. This is the second phase, also known as the backpropagation process. The detailed derivation of the Levenberg–Marquardt algorithm can be found in previous literature [35,36].

To avoid overfitting and increase the generalization capability of the trained ANN, an early stopping technique is utilized during the training procedure. The entire dataset is divided into three subsets, namely, the training set, the validation set and the testing set. During the training procedure, the first two datasets are used. The training set is used for computing and updating the network weights and biases. During the training process, as with the training error, the model performance on the validation set is simultaneously monitored. When the validation error starts to increase for a certain number of iterations (set as 10 here), the training is terminated. The increase in the validation error indicates the decrease in the model generalization ability. The entire network will then return the weight and bias matrixes when the overall error is at its minimum.

### 2.1. Input and Output Variables

**Figure 2:**
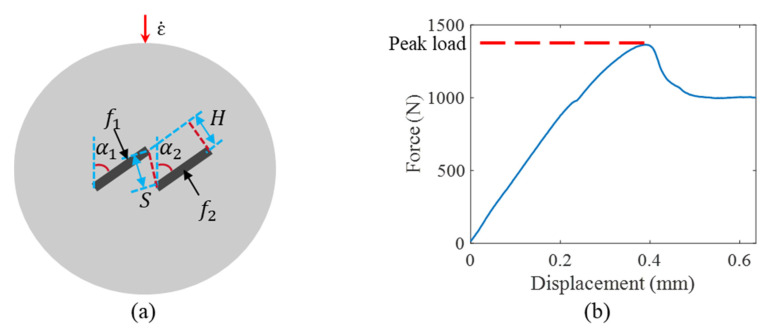
(**a**) The schematic of an overlapped flaw configuration with input variables labeled; (**b**) the output (peak load) is extracted from a typical force–displacement curve.

The current study aims to investigate the ANN application in establishing the relationship between the rock strength (peak load) and rock heterogeneous factors at different strain rates. An experimental dataset including 84 Brazilian disc experiments has been collected from our previous studies [31,32,33]. For the multiple-crack system, descriptive and appropriate crack geometric parameters should be properly selected, which is vital to obtain an acceptable predictive accuracy with a relatively small dataset. Herein, the selected parameters include (1–2) the crack inclination angle with loading axis $\alpha_{1}$, $\alpha_{2}$, where the subscripts are used to distinguish the different flaws; (3) the crack offset distance H; (4) the crack tip distance S, (5–6) the flaw filling $f_{1}$, $f_{2}$, and (7) the strain rate $\dot{\varepsilon }$, as shown in Figure 2a. These seven parameters construct the input variables, and the peak load *PL*, as marked in Figure 2b, is the output variable. The entire dataset containing 84 experiments is listed in Table A1 in Appendix A. For the single flaw tests, H and S are equal to 0. If the open flaw is filled, its corresponding filling parameter is set as 1; otherwise, it will be 0.

The raw dataset can be normalized on a scale of 0 to 1 to improve the network performance. The normalization equation is given as follows:

(3) $$x_{i}^{N}=\frac{x_{i}^{O}-x^{min}}{x^{max}-x^{min}}$$

where $x_{i}^{N}$ and $x_{i}^{O}$ are the *i*th normalised and original variable values, respectively. $x^{min}$ and $x^{max}$ are the minimal and maximum values of the variable set.

### 2.2. Optimization of ANN

In this application, the trial-and-error method is adopted for optimizing the network structure and operational parameters because of the small dataset and low computational consumption for each trial [27]. Different transfer function combinations and the min–max normalization effect are tuned in order to find the best setting. Meanwhile, a parametric study for hidden layer neuron number is conducted.

For better comparison, different ANNs are required to be performed on the same training, validation and testing dataset divisions, accounting for 60, 12 and 12 out of the total 84 experiments, respectively. To be specific, the validation dataset contains Test No. 5, 13, 15, 23, 32, 42, 49, 53, 58, 64, 76, 82, the testing dataset contains the Test No. 6, 12, 17, 25, 35, 37, 44, 52, 62, 68, 70, 80, while the rest belongs to the training dataset. Since the network is trained to start from different initial weights and biases, this may lead to different solutions even for the same dataset division. Each ANN structure is trained ten times and the ANN with the best performance is selected. The *MSE* or *R* values for the training and testing dataset are chosen to evaluate the training and generalization performances, respectively. The ANN, owing the smaller average *MSE* or the larger average *R*, is considered to have better performance.

#### 2.2.1. Transfer Functions

**Figure 3:**
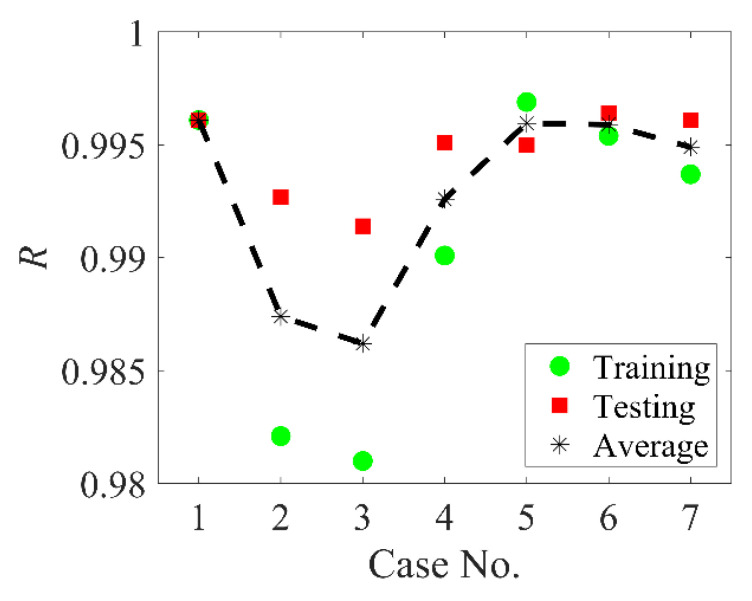
The performances of ANNs with different transfer functions and normalization settings.

Based on the previous studies [28,37], one hidden layer is applied due to the limited input and output variables. The network with too many hidden layers or neurons can be easily over-trained and will not sufficiently predict the new data. The detailed analysis of the neuron number contained in the hidden layer is elaborated in the subsequent section. The different setting combinations are performed on the same 7-10-1 ANN, where the ANN contains 10 hidden layer neurons. Table 1 lists seven different transfer functions and normalization setting combinations. Figure 3 presents the regression values for different ANN settings.

As shown in Figure 3, the setting of case 1 is superior to other cases and the following study adopted same transfer functions and normalization setting. It should also be noted that, for cases 2, 3 and 4, the trained ANNs present a better testing accuracy than the training results, which may result from the fixed data set splitting here. Although these three cases have poor training accuracy for the training dataset, the trained ANNs appear to be more suitable for the testing dataset, leading to a higher regression value [27].

#### 2.2.2. Hidden Layer Neuron Number

**Figure 4:**
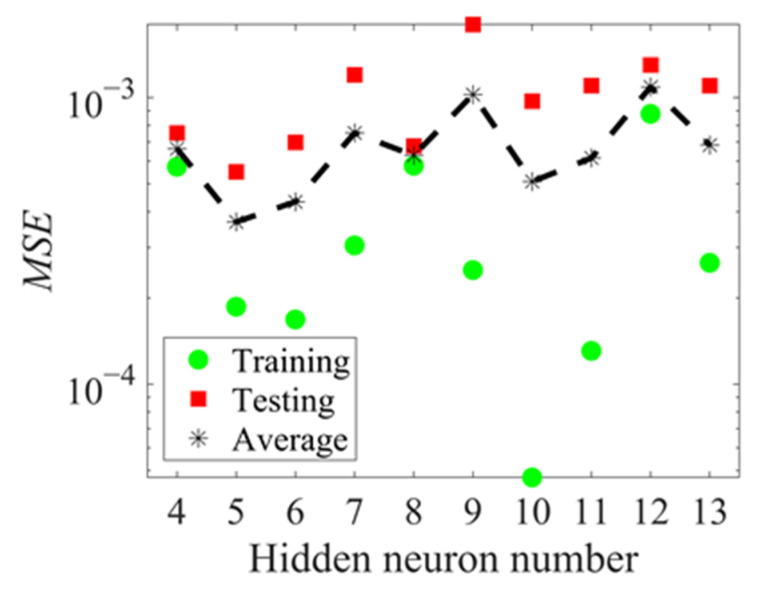
Performance of ANNs with different hidden neuron numbers.

Yan et al. [28] reported the following formulation to estimate the neuron number in the single hidden layer:

(4) $$m=\sqrt{n+l}+a$$

where m, n and l the neuron numbers of hidden, input and output layers, respectively. a is a constant, ranging from 1 to 10. The neurons number in the hidden layers is investigated in the range of 4 to 13. The comparison between different ANNs is also performed for the same abovementioned dataset divisions and the *MSE* values for each case are shown in Figure 4. The training *MSE* is usually smaller than the testing *MSE*. The neuron number in the hidden layer is chosen to be 5 since this leads to a relatively smaller average *MSE* value.

### 2.3. ANN Training

Table 2 provides the ANN setting after optimization. The training is repeated 10 times and the ANN with the best performance is selected.

**Figure 5:**
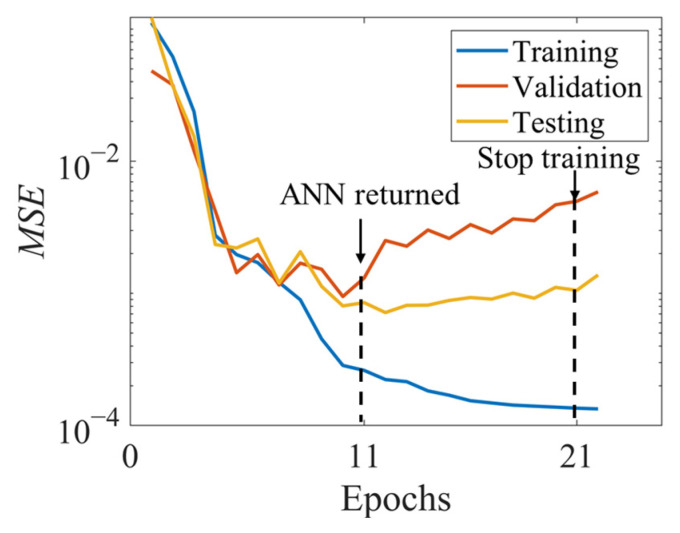
The typical training process of ANN.

**Figure 6:**
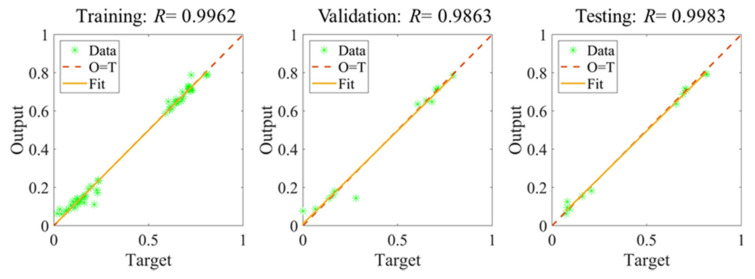
The regression values of the trained ANN.

The training procedure of the picked ANN is illustrated in Figure 5. After epoch-11, the error of the validation starts to increase, as well as that of the testing dataset. Although the training accuracy is still increasing, the generalization ability tends to decrease. As a result, the training stops at epoch-21 and returns the training value at epoch-11 due to the early stopping setting. The outcome of the chosen ANN is shown in Figure 6. It is observed that the *R* values for training, validation and testing are 0.9962, 0.9863, and 0.9983, respectively. The overall regression value *R* is 0.9951.

The weights and biases associated with this trained ANN are given as follows:

$$\begin{array}{l} \left[IW1\right]=\left[\begin{array}{lllllll} 1.4976 & \;0.5617 & \;0.5583 & \;0.0933 & \;0.4677 & \;-0.6805 & \;-0.4803\\ 0.4817 & \;-0.5290 & \;-0.9578 & \;0.5081 & \;0.6548 & \;-0.2266 & \;0.5202\\ 0.7050 & \;0.5152 & \;0.3833 & \;-0.1897 & \;1.2370 & \;0.0207 & \;-2.2187\\ -0.5221 & \;0.2058 & \;0.9680 & \;1.1084 & \;0.0890 & \;-0.2536 & \;1.1518\\ 0.2287 & \;-0.6957 & \;-0.3462 & \;0.4682 & \;0.8604 & \;-0.0799 & \;-2.0323 \end{array}\right]\\ {\left[IW2\right]}^{T}=\left[\begin{array}{ccc} 0.3040 & 0.6847 & -0.7211 \end{array}\begin{array}{cc} \;0.5353 & 0.1036 \end{array}\right]\\ {\left[b1\right]}^{T}=\left[\begin{array}{ccc} -1.5586 & -0.9090 & 0.0191 \end{array}\begin{array}{cc} \;-1.4385 & 1.2869 \end{array}\right]\\ {\left[b2\right]}^{T}=\left[0.6196\right] \end{array}$$

## 3. Results and Discussion

The entire dataset is used to evaluate the accuracy of the predicted peak load by the ANN approach. All predicted results by the trained ANN are listed in Appendix A. The importance factors of each input variable on the peak load are calculated by the sensitivity analysis.

### 3.1. Predicting Results

**Figure 7:**
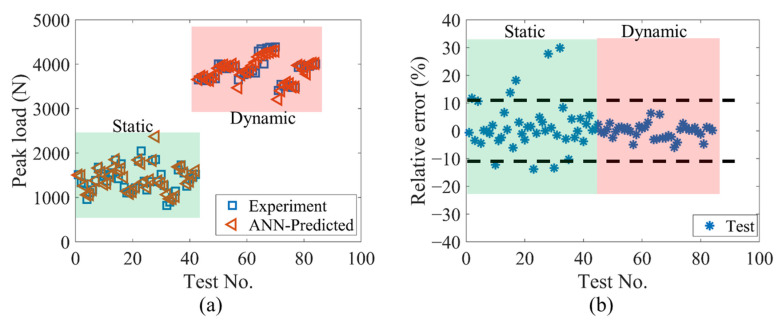
(**a**) The comparison of predicted and experimental results; (**b**) the corresponding relative error for each test.

Figure 7a presents a comparison of the peak loads predicted by the ANN model with the experimental results, and Figure 7b shows the relative error values between the ANN-predicted and experimental results. The relative error is calculated as $\left(PL_{ANN}-PL_{Experiment}\right)/PL_{Experiment}$, where $PL_{ANN}$ and $PL_{Experiment}$ are the ANN predicted and experimental results, respectively. Meanwhile, the statistical analysis based on the ratio of $PL_{ANN}/PL_{Experiment}$ and mean absolute relative error is performed, and the key descriptive statistics are summarized in Table 3.

It is clear that the dynamic test section has better predicted results than the static test section in terms of mean, standard deviation (SD) and coefficient of variation (COV) for the ratio of $PL_{ANN}/PL_{Experiment}$, as well as a smaller mean absolute relative error. Except for several data points in the static test section, the majority of the predicted data have a relative error within ±10%.

Generally speaking, the ANN model demonstrates its strong ability to quickly (within seconds) and accurately predict the peak load. The current trained ANN model has a good mean value (close to 1) and a small standard deviation, indicating that the ANN model has a good generalization ability. In addition, the relatively small COV value indicates fewer scattering results. The overall mean absolution relative error is only 4.49%.

### 3.2. Sensitivity Analysis

To quantify the relative influence factor of each input variable on the peak load, Garson’s equation, derived from neuron weight matrixes (*IW1* and *IW2*), is applied, as illustrated below [38,39]:

(5) $$I_{ik}=\frac{\sum_{j=1}^{L}\left(\left(\frac{\left|IW1_{ij}\right|}{\sum_{i=1}^{N}\left|IW1_{ij}\right|}\right)\times \left|IW2_{jk}\right|\right)}{\sum_{i=1}^{N}\left(\sum_{j=1}^{L}\left(\left(\frac{\left|IW1_{ij}\right|}{\sum_{i=1}^{N}\left|IW1_{ij}\right|}\right)\times \left|IW2_{jk}\right|\right)\right)}$$

where $I_{ik}$ is the relative importance factor of the *i*th input neuron on the *k*th output neuron, *N* and *L* are the numbers of input and hidden neurons, respectively, $IW1_{ij}$ is the connection weight matrix between the *i*th input neuron and the *j*th hidden neuron, while $IW2_{jk}$ is the connection weight matrix between the *j*th hidden neuron and the *k*th output neuron. Note that the ANN model is obtained based on a series of min–max normalized inputs and output data.

**Figure 8:**
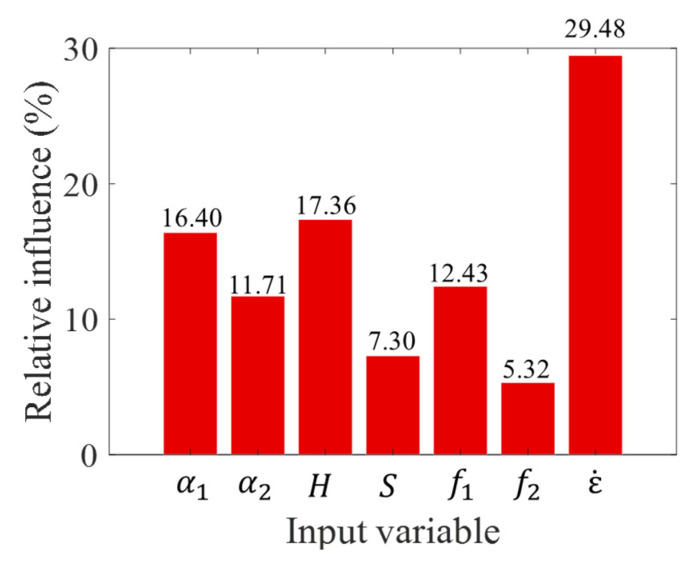
The sensitivity importance of input variables.

Figure 8 shows the relative influence of each individual input variable. The results of the analysis illustrate that the strength of heterogeneous rock is greatly affected by all considered input variables, while the strain rate $\dot{\varepsilon }$ is the most predominant variable affecting the rock strength in this study.

The effects of input number are further evaluated by comparing the mean absolute relative error derived from the entire dataset. The results for different selections of inputs are summarized in Table 4. Note that all ANN models with different inputs are trained under the same ANN setting listed in Table 2. It can be observed from Table 2 that the reduction in the inputs will decrease the overall performance of the ANN models in the current study. In particular, the strain rate $\dot{\varepsilon }$ cannot be ignored here because of the large prediction error presented in Case 8, which also validates the sensitivity analysis results. The error difference among other reported cases is, to some extent, not obvious.

## 4. Conclusions

The present study shows that the artificial neural network (ANN) approach can be applied to predict the influences of heterogeneity on rock strength at different strain rates. In the current study, seven input variables (initial crack inclination angle $\alpha_{1}$, $\alpha_{2}$; relative position H, S; filling material $f_{1}$, $f_{2}$ and strain rate $\dot{\varepsilon }$) are considered and only one output variable, peak load PL. The best prediction performance of the ANN is observed when using the tan-sigmoid transfer functions in both the hidden and output layers, as well as the five neurons contained in the single hidden layer. Although only a limited number of experiment tests are implemented, the trained ANN presents a fast and accurate strength predicting performance, as reflected in the overall regression value of 0.9951. Finally, the sensitivity study for the input variables determines that strain rate is the most important factor affecting the mechanical performance of heterogeneous rocks. In the practical application process, the rock strength can be forecasted by several simple and measurable physical variables. In conclusion, the strong capacity for rapid convergence and high predictive accuracy of the ANN model is corroborated.

Limited by current experimental tests, the input variable pairs, namely $\alpha_{1}$, $\alpha_{2}$ and $f_{1}$, $f_{2}$, both have the same values. Therefore, the applicability of the trained ANN model for more complex conditions concerning flaws with diverse crack inclination angles and filling material needs to be further validated by corresponding experiments. Because the ANN approach has the inherent advantage of being able to be extended (in terms of both depth and width), it possesses promising potential for establishing a more complex nonlinear relationship between the rock strength and rock heterogeneity when more heterogeneous physical quantities are involved. However, from a practice point of view, further systematic sampling with variations poses a great challenge for the large quantity of high-quality laboratory-tested samples, which may be problematic for some special fragile or heavily weathered rocks, as mentioned above. Hence, the proposed ANN approach, to some extent, can minimize the number of unnecessary experiments in order to ensure the rapid estimation of rock mechanical properties, which provides an alternative means of handling complex rock engineering problems. At the same time, with the aid of the more complete database, the reliability of the proposed ANN model can be further validated. It is worth noting that the current ANN approach cannot provide detailed information on failure patterns, such as those in experiments and simulations, and further research is deemed necessary.

## Figures and Tables

**Table 1:** Combinations of different transfer functions and normalization settings.

Case No.	Input Variables	Hidden Layer	Output Layer	Output Variable
1	Normalized	Tan-sigmoid	Tan-sigmoid	Normalized
2	Normalized	Tan-sigmoid	Sigmoid	Normalized
3	Normalized	Sigmoid	Sigmoid	Normalized
4	Normalized	Tan-sigmoid	Linear	Raw
5	Normalized	Sigmoid	Linear	Raw
6	Raw	Tan-sigmoid	Linear	Raw
7	Raw	Sigmoid	Linear	Raw

**Table 2:** The ANN setting for predicting peak load.

Input layer neuron number	7
Hidden layer neuron number	5
Output layer neuron number	1
Data division	Randomly (70% training, 15% validation, 15% testing)
Hidden layer transfer function	Tan-sigmoid
Output layer transfer function	Tan-sigmoid
Early stopping criterion	10 epochs for validation check
Target error	10^−6^
Training algorithm	Levenberg–Marquardt

**Table 3:** Descriptive statistics of the peak loads predicted by the ANN approach.

Test No.	$PL_{ANN}/PL_{Experiment}$			Mean Absolute Relative Error (%)
	Mean	SD	COV (%)	
Static (No.1–No.42)	1.01	0.12	1.37	7.46
Dynamic (No.43–No.84)	1.00	0.02	0.04	1.51
Overall (No.1–No.84)	1.00	0.08	0.70	4.49

**Table 4:** Effects of input number on the prediction by ANN models.

Case No.	Number of Inputs	Inputs	Mean Absolute Relative Error (%)
1	7	$\alpha_{1}$, $\alpha_{2}$, H, S,$\;f_{1}$, $f_{2}$, $\dot{\varepsilon }$	4.49 (Reference)
2	6	$\alpha_{2}$, H, S,$\;f_{1}$, $f_{2}$,$\;\dot{\varepsilon }$	6.26
3		$\alpha_{1}$,H, S,$\;f_{1}$, $f_{2}$,$\;\dot{\varepsilon }$	5.75
4		$\alpha_{1}$,$\alpha_{2}$,S,$\;f_{1}$, $f_{2}$,$\;\dot{\varepsilon }$	7.39
5		$\alpha_{1}$,$\alpha_{2}$,H,$f_{1}$, $f_{2}$,$\;\dot{\varepsilon }$	6.02
6		$\alpha_{1}$,$\alpha_{2}$,H, S,$\;f_{2}$,$\;\dot{\varepsilon }$	5.96
7		$\alpha_{1}$,$\alpha_{2}$,H, S,$\;f_{1}$,$\;\dot{\varepsilon }$	6.36
8		$\alpha_{1}$,$\alpha_{2}$,H,S,$\;f_{1}$,$f_{2}$	68.40
9	5	$\alpha_{1}$,H,$\;f_{1}$,$f_{2}$,$\;\dot{\varepsilon }$	6.38
10		$\alpha_{1}$, H,S,$\;f_{1}$,$\;\dot{\varepsilon }$	6.43
11		$\alpha_{1}$,$\alpha_{2}$,H,$f_{1}$,$\;\dot{\varepsilon }$	6.50
12	4	$\alpha_{1}$, H,$\;f_{1}$,$\;\dot{\varepsilon }$	6.10

## Data Availability

The data presented in this study are available on request from the corresponding author.

## Appendix A

**Table A1:** Summarization of the experimental raw dataset prepared for ANN and trained ANN predicted results.

Test No.	Input							Output	
	Initial Crack Inclination Angle from Loading Axis (^o^)		Relative Position (mm)		Filling		Strain Rate (s^−1^)	Peak Load (N)	
								Experiment	ANN
	$\alpha_{1}$	$\alpha_{2}$	H	S	$f_{1}$	$f_{2}$	$\dot{\varepsilon }$	PL	*PL*
1	90	90	0	0	0	0	0.0001	1515	1486.098
2	75	75	0	0	0	0	0.0001	1333	1326.732
3	60	60	0	0	0	0	0.0001	1306	1225.101
4	45	45	0	0	0	0	0.0001	956	1196.695
5	30	30	0	0	0	0	0.0001	1109	1204.291
6	15	15	0	0	0	0	0.0001	1169	1238.981
7	0	0	0	0	0	0	0.0001	1380	1319.490
8	90	90	0	0	1	1	0.0001	1678	1725.743
9	75	75	0	0	1	1	0.0001	1533	1523.316
10	60	60	0	0	1	1	0.0001	1476	1444.671
11	45	45	0	0	1	1	0.0001	1369	1445.347
12	30	30	0	0	1	1	0.0001	1520	1494.781
13	15	15	0	0	1	1	0.0001	1557	1610.455
14	0	0	0	0	1	1	0.0001	1843	1849.571
15	90	90	0	7.334	0	0	0.0001	1423	1443.698
16	75	75	0	7.334	0	0	0.0001	1756	1305.584
17	60	60	0	7.334	0	0	0.0001	1230	1195.725
18	45	45	0	7.334	0	0	0.0001	1105	1159.104
19	30	30	0	7.334	0	0	0.0001	1101	1164.367
20	15	15	0	7.334	0	0	0.0001	1169	1200.831
21	0	0	0	7.334	0	0	0.0001	1217	1288.699
22	90	90	0	7.334	1	1	0.0001	1816	1643.400
23	75	75	0	7.334	1	1	0.0001	2049	1453.946
24	60	60	0	7.334	1	1	0.0001	1365	1367.216
25	45	45	0	7.334	1	1	0.0001	1171	1368.303
26	30	30	0	7.334	1	1	0.0001	1354	1428.184
27	15	15	0	7.334	1	1	0.0001	1832	1568.657
28	0	0	0	7.334	1	1	0.0001	1856	1855.662
29	90	90	5.555	5.686	0	0	0.0001	1359	1377.247
30	75	75	5.555	5.686	0	0	0.0001	1522	1346.187
31	60	60	5.555	5.686	0	0	0.0001	1270	1262.478
32	45	45	5.555	5.686	0	0	0.0001	819	1154.207
33	30	30	5.555	5.686	0	0	0.0001	894	1096.502
34	15	15	5.555	5.686	0	0	0.0001	1002	1083.879
35	0	0	5.555	5.686	0	0	0.0001	1140	1093.918
36	90	90	5.555	5.686	1	1	0.0001	1620	1665.344
37	75	75	5.555	5.686	1	1	0.0001	1732	1623.381
38	60	60	5.555	5.686	1	1	0.0001	1554	1489.184
39	45	45	5.555	5.686	1	1	0.0001	1256	1379.604
40	30	30	5.555	5.686	1	1	0.0001	1419	1366.166
41	15	15	5.555	5.686	1	1	0.0001	1456	1417.751
42	0	0	5.555	5.686	1	1	0.0001	1518	1526.933
43	90	90	0	0	0	0	350	3650	3663.233
44	75	75	0	0	0	0	350	3700	3617.357
45	60	60	0	0	0	0	350	3630	3620.612
46	45	45	0	0	0	0	350	3655	3639.233
47	30	30	0	0	0	0	350	3670	3658.648
48	15	15	0	0	0	0	350	3697	3677.001
49	0	0	0	0	0	0	350	3675	3695.113
50	90	90	0	0	1	1	350	4010	3925.057
51	75	75	0	0	1	1	350	3980	3916.149
52	60	60	0	0	1	1	350	3930	3929.861
53	45	45	0	0	1	1	350	3900	3949.114
54	30	30	0	0	1	1	350	3945	3969.769
55	15	15	0	0	1	1	350	3955	3991.496
56	0	0	0	0	1	1	350	3950	4014.363
57	90	90	0	7.334	0	0	350	3650	3724.057
58	75	75	0	7.334	0	0	350	3810	3665.866
59	60	60	0	7.334	0	0	350	3768	3679.004
60	45	45	0	7.334	0	0	350	3795	3726.472
61	30	30	0	7.334	0	0	350	3825	3781.462
62	15	15	0	7.334	0	0	350	3850	3836.380
63	0	0	0	7.334	0	0	350	3805	3889.384
64	90	90	0	7.334	1	1	350	4300	4268.950
65	75	75	0	7.334	1	1	350	4350	4270.513
66	60	60	0	7.334	1	1	350	4005	4279.144
67	45	45	0	7.334	1	1	350	4365	4288.077
68	30	30	0	7.334	1	1	350	4386	4295.510
69	15	15	0	7.334	1	1	350	4370	4301.435
70	0	0	0	7.334	1	1	350	4390	4306.144
71	90	90	5.555	5.686	0	0	350	3410	3400.248
72	75	75	5.555	5.686	0	0	350	3550	3521.862
73	60	60	5.555	5.686	0	0	350	3510	3487.539
74	45	45	5.555	5.686	0	0	350	3500	3492.108
75	30	30	5.555	5.686	0	0	350	3520	3547.000
76	15	15	5.555	5.686	0	0	350	3480	3610.143
77	0	0	5.555	5.686	0	0	350	3475	3667.492
78	90	90	5.555	5.686	1	1	350	3930	4020.174
79	75	75	5.555	5.686	1	1	350	3975	3946.840
80	60	60	5.555	5.686	1	1	350	3910	3964.291
81	45	45	5.555	5.686	1	1	350	3970	3980.738
82	30	30	5.555	5.686	1	1	350	3955	3982.104
83	15	15	5.555	5.686	1	1	350	3980	3936.657
84	0	0	5.555	5.686	1	1	350	4010	3932.417
